# Role of Non-Covalent Interactions in Carbonic Anhydrase I—Topiramate Complex Based on QM/MM Approach

**DOI:** 10.3390/ph16040479

**Published:** 2023-03-23

**Authors:** Kamil Wojtkowiak, Aneta Jezierska

**Affiliations:** Faculty of Chemistry, University of Wrocław, ul. F. Joliot-Curie 14, 50-383 Wrocław, Poland

**Keywords:** Carbonic anhydrase I, Topiramate (TPM), non-covalent interactions, QM/MM, DFT, IGMH, IRI, QTAIM, NBO, SAPT

## Abstract

Carbonic anhydrase (CA) I with a Topiramate (TPM) complex was investigated on the basis of a Quantum Mechanics/Molecular Mechanics (QM/MM) approach. The QM part was treated using Density Functional Theory (DFT) while the MM was simulated using Amberff14SB and GAFF force fields. In addition, the TIP3P model was applied to reproduce the polar environment influence on the studied complex. Next, three snapshots (after 5 ps, 10 ps, and 15 ps of the simulation time) were taken from the obtained trajectory to provide an insight into the non-covalent interactions present between the ligand and binding pocket of the protein. Our special attention was devoted to the binding site rearrangement, which is known in the literature concerning the complex. This part of the computations was performed using ωB97X functional with Grimme D3 dispersion corrections as well as a Becke–Johnson damping function (D3-BJ). Two basis sets were applied: def2-SVP (for larger models) and def2-TZVPD (for smaller models), respectively. In order to detect and describe non-covalent interactions between amino acids of the binding pocket and the ligand, Independent Gradient Model based on Hirshfeld partitioning (IGMH), Interaction Region Indicator (IRI), Quantum Theory of Atoms in Molecules (QTAIM) and Natural Bond Orbitals (NBO) methods were employed. Finally, Symmetry-Adapted Perturbation Theory (SAPT) was applied for energy decomposition between the ligand and protein. It was found that during the simulation time, the ligand position in the binding site was preserved. Nonetheless, amino acids interacting with TPM were exchanging during the simulation, thus showing the binding site reorganization. The energy partitioning revealed that dispersion and electrostatics are decisive factors that are responsible for the complex stability.

## 1. Introduction

Non-covalent interactions are one of the most important interplay in the whole of biology and chemistry [1,2,3,4,5]. A relatively low stabilizing effect is compensated for by their ubiquity [6,7]. It is undoubtedly known that the so-called conventional hydrogen bonds, in which the proton donor and acceptor atoms are usually oxygens or nitrogens, have a huge impact on the secondary and tertiary structures of proteins, as well as on the secondary structure of the nucleic acids [8,9]. The usual hydrogen bond definition is presented as: X-H…:Y, which is the interaction between a hydrogen atom attached to an electronegative atom (X) with species having a lone electron pair (Y). As it was mentioned earlier, in the biological systems, X, as well as Y, are usually oxygen or nitrogen atoms, with sulfur or halogens being less common. Nonetheless, as we will argue in this paper, the impact of “weak hydrogen bonds” can also be of great importance, especially in the context of ligand–protein interactions and crystal packing [10,11,12]. The weak hydrogen bonds can be classified by the presence of the weak donors, such as: C-H or P-H; weak acceptors such as: organic halogens, C-X (X = F, Cl, or Br), S, Se, Te, or C atoms; or aromatic frameworks. The first conclusive experimental evidence of the presence of C-H…O, C-H…N and C-H…Cl interactions was provided by Taylor and Kennard in 1982 [13]. This type of interaction is usually characterized by 3.0–3.8 Å and 2.0–2.8 Å distances of donor–acceptor and proton–acceptor species. The calculations indicated that the most important factor affecting the energy and length of this type of hydrogen bonding is the acidity of the C-H donors (bond energies are typically below 4 kcal/mol). In fact, the acidity depends on the hybridization of the carbon atom (it grows as the hybridization changes in a row: sp^3^, sp^2^, sp) [11].

**Figure 1:**
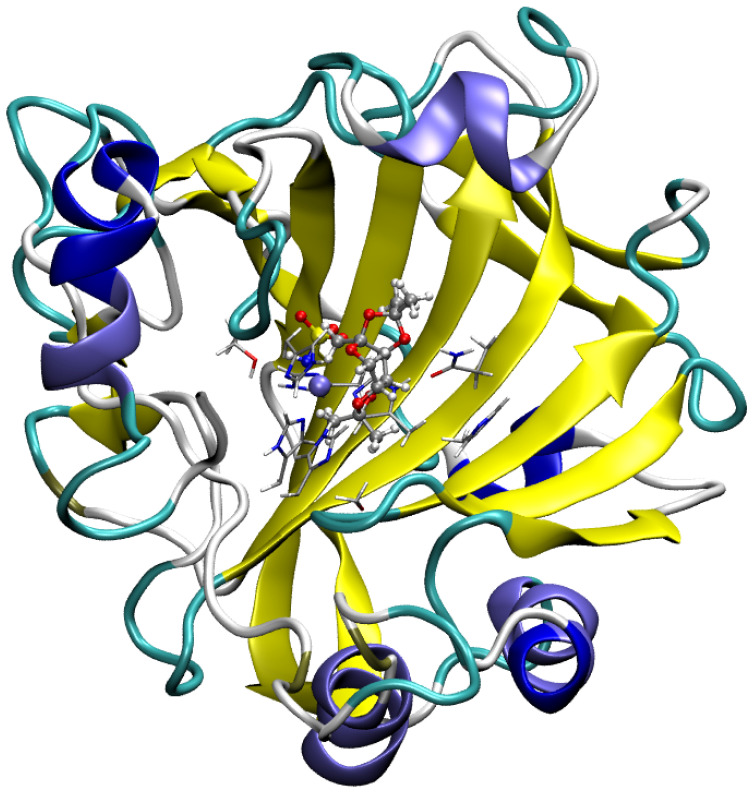
Graphical representation of the model used in the QM/MM molecular dynamics simulations (MM and QM water molecules have been omitted for clarity). The binding site and Topiramate are represented using the CPK model (this part was treated at the QM level), while the rest of the protein (treated at the MM level) was visualized with the cartoon representation. Color coding: white—hydrogen, cyan—carbon, red—oxygen, blue—nitrogen, yellow—sulfur, and purple—zinc.

In the current study, we investigate the Carbonic anhydrase I (CA I) which is a member of a family of metalloenzymes and is involved in diverse physiological processes such as the secretion of gastric or pancreatic juices, pH regulation, and ion transport as well as bone resorption [14,15,16,17,18]. The main role of CA isozymes in the organism of the host is the reversible catalysis of the CO_2_ hydration, which produces the bicarbonate and hydronium ions [19]. Various isozymes have been identified in different parts of the body, whereas Carbonic anhydrase II (CA II) was found in the wide variety of cells, such as: cartilage, liver, lung, skeletal muscles, brain and pancreas. Other isoforms distribution is more limited—CA I is a cytosolic form of CA and is found mainly in the erythrocytes and GI tract [20,21]. Due to the highly conserved structure of the CA isozymes catalytic domains, the analysis regarding non-covalent interactions present in the active-site of CA I can be also extrapolated (to some extent) to other isoforms of Carbonic anhydrase [22,23]. Drug design of the CA inhibitors (CAIs) that are isozyme-selective is an important endeavor, due to ubiquity of various CA isozymes in the human organism—CAIs could be used as antitumor or antiobesity agents as well as diagnostic tools [18]. One of the CAIs is Topiramate (TPM), sulfamate-substituted monosaccharide—the drug already present on the market, used for the treatment of migraine and epilepsy [24,25]. Nonetheless, TPM is finding more and more applications and its efficacy toward idiopathic intracranial hypertension, bulimia nervosa, obsessive-compulsive disorder and bipolar disorder was recently examined [26,27,28,29]. In the current work, we discuss in detail the mode of binding and the impact of the non-covalent interactions on the stability of the TPM molecule in the active site of the Carbonic anhydrase I from the perspective of the static and dynamic methods of quantum chemistry. We performed hybrid Quantum Mechanics/Molecular Mechanics (QM/MM) [30] simulations (the studied system is presented in Figure 1). To simulate the key part of the complex (QM—binding pocket with the ligand), also taking into account the impact of the remaining part of the protein (that imposes profound rigidness on the amino acids involved in interactions with the ligand—MM).

**Figure 2:**
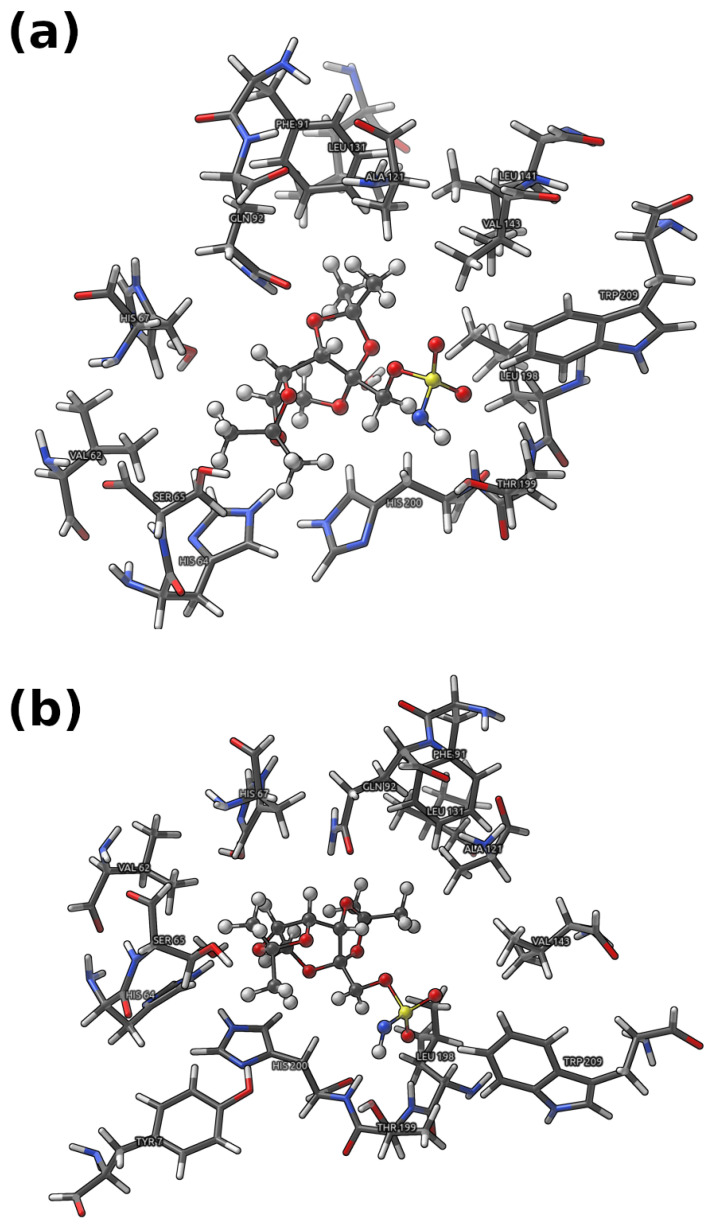
Graphical representation of the large model used in the present study. (**a**) denotes model analyzed at 5 and 10 ps (only atoms positions with respect to the ligand changed—all taken amino acids were the same for 5 and 10 ps; visualization is based on the atoms configuration taken at 5 ps of the simulation time), whereas (**b**) denotes model obtained as a third snapshot (15 ps) of the QM/MM run. The amino acids in the binding site are presented using licorice while the ligand—Topiramate using CPK. Color coding: white—hydrogen, grey—carbon, red—oxygen, blue—nitrogen, and yellow—sulfur.

**Figure 3:**
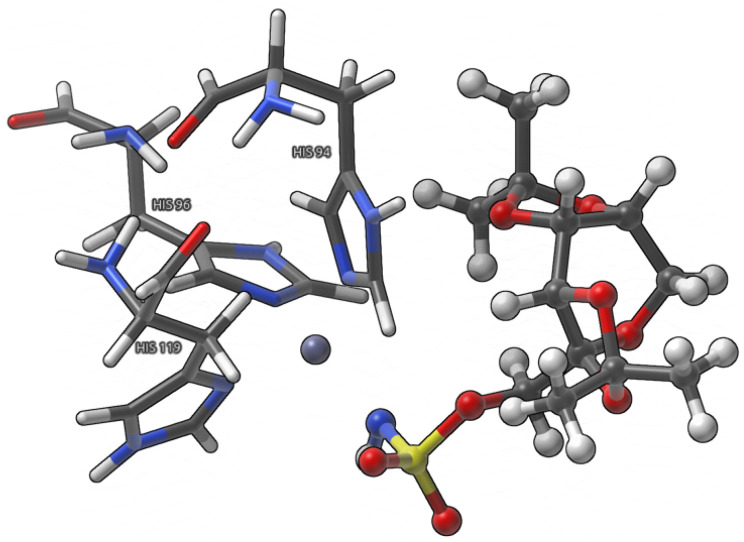
Graphical representation of the small model used in the study. Amino acids coordinating the metal ion are presented using licorice visualization, whereas the ligand—Topiramate as CPK model. Color coding: white—hydrogen, grey—carbon, red—oxygen, blue—nitrogen, yellow—sulfur, and purple—zinc.

The obtained results shed a new light onto an important subject of CA I interactions and the mode of binding of the Topiramate-CA I complex and will facilitate the rational design of the inhibitors targeted at the family of the Carbonic anhydrase enzymes. The detailed study of non-covalent interactions and dative covalent bonds of the Topiramate-CA I binding site was facilitated using IGMH [31,32], QTAIM [33], NBO [34] and IRI [35] methods. Some of the approaches were proposed quite recently and they enabled us to provide a more accurate description of intra- and intermolecular interactions based on electron density. In our case, the methods are useful to define and characterize the interplays present in the studied systems. The above-mentioned frameworks were applied to:

(i) Large model (Figure 2), where the interactions between Topiramate (TPM) and the binding site, excluding the ones involved in the coordination of the zinc ion, were studied;
(ii) Small model (Figure 3), where the interactions between Topiramate (TPM) and amino acids involved in zinc coordination, as well as the zinc coordination itself, were analyzed.

**Figure 4:**
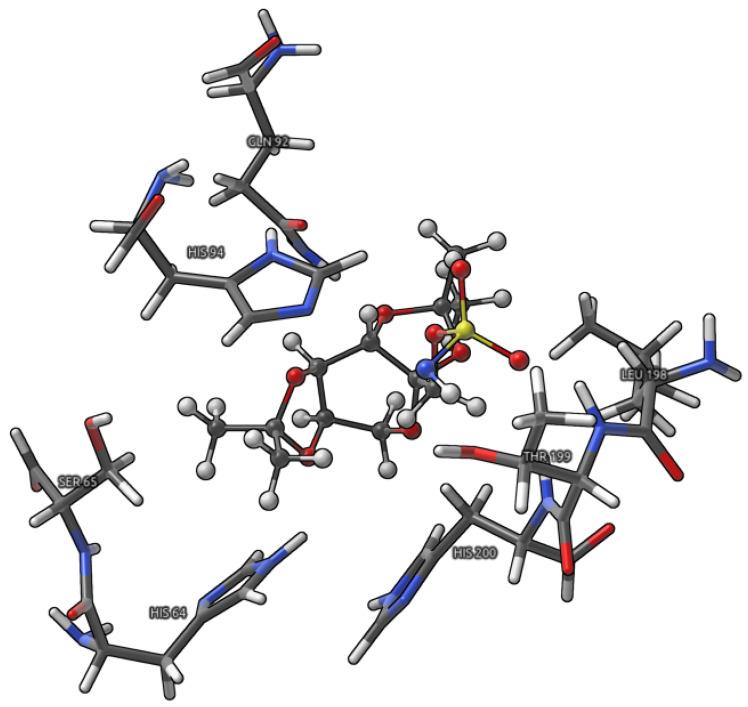
Graphical representation of the model used for SAPT energy decomposition. Topiramate is presented as CPK model, whereas amino acids of the binding site as licorices. Color coding: white—hydrogen, grey—carbon, red—oxygen, blue—nitrogen, and yellow—sulfur.

The interaction energy estimation and its decomposition in the Symmetry-Adapted Perturbation Theory (SAPT) [36] scheme was performed for model (see Figure 4) chosen on the basis of the electron density (ED) analyses.

**Figure 5:**
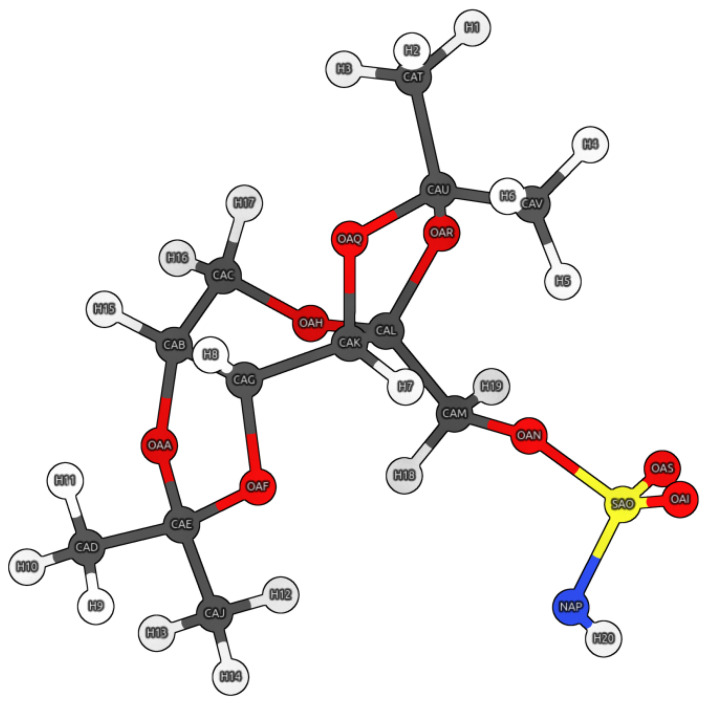
Graphical representation of the Topiramate (TPM) molecule and its atom designations. Color coding: white—hydrogen, grey—carbon, red—oxygen, blue—nitrogen, and yellow—sulfur.

Each studied model was extracted at a specified instant of time during the course of QM/MM MD. The Topiramate (TPM) ligand with atom designations is presented in Figure 5.

To our knowledge, this is the first work that combines the QM/MM formalism with a very detailed analysis of the binding site with special attention to the reorganization of interactions at the binding site based on electron density approaches.

## 2. Results and Discussion

### 2.1. Analysis of Non-Covalent Interactions Present in a Large Model

**Figure 6:**
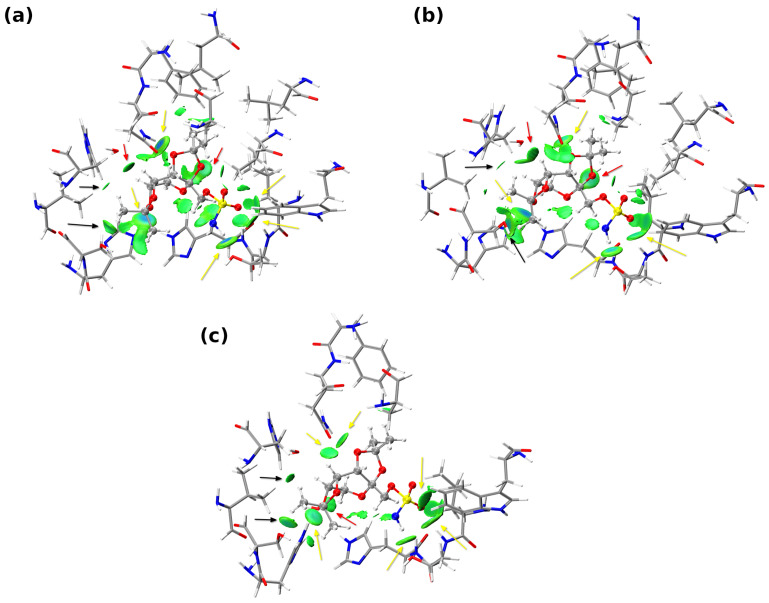
Colored isosurfaces (in the ED range of: −0.05 < sign(λ_2_)ρ < 0.05 a.u.) of *∂*g = 0.005 a.u. for the large model of the binding site of CA I. Structures were taken from the course of QM/MM Molecular Dynamics. (**a**) snapshot at 5 ps, (**b**) snapshot at 10 ps, and (**c**) snapshot at 15 ps. Ligand atoms are depicted using the CPK representation, whereas amino acids of the binding site are visualized as licorices. Color coding: grey—carbon, white—hydrogen, red—oxygen, blue—nitrogen, and yellow—sulfur. Yellow arrows point to the isosurfaces that indicate the most pronounced interactions with the protein, while red arrows indicate the presence of interactions with water molecules.

Comprehensive and detailed analysis of the non-covalent interactions between the ligand (TPM) and the neighboring amino acids of the CA I was enabled by using the IGMH [31,32], QTAIM [33], and NBO [34] theories. The Independent Gradient Model based on Hirshfeld partitioning (IGMH) was used to illustrate all the existing interatomic interactions between fragments. The ligand molecule was chosen as fragment 1, whereas the others, two water molecules and amino acids, were treated as fragment 2. The central quantity in the IGMH is g^IGM^ (the sum of the absolute values of the electron density (ED) gradients of different atoms), the so-called density gradient of non-interacting system, that serves as the upper limit of g. Subsequently, by subtracting the g (the absolute value of the sum of ED gradients of different atoms), one can efficiently reveal the interfragment interactions. Whereas the original formulation of IGM [31] was defined using the densities of atoms in their free states (promolecular approximation). In the current study, we have chosen to link the atom densities with the actual densities obtained from quantum chemical calculations via the Hirshfeld partitioning [32]. The IGMH analysis results for three snapshots of the QM/MM MD are presented in Figure 6. The yellow arrows indicate isosurfaces corresponding to the most stabilizing interactions between the TPM ligand and the binding site. It should be emphasized that, due to the fact that the analyzed structures were not in their minima at the potential energy surface, some of *∂*g isosurfaces may sometimes falsely indicate the presence of attractive interactions—consider light green to even partly blueish isosurfaces (indicated by black arrows) between the CH_2_ of HIS67 and SER65 with the CH_3_ of TPM (present in each snapshot). While certainly not attractive in nature, these contacts also have a non-negligible impact on the ligand position in the binding site.

Regarding the non-covalent interactions: one can observe the blueish isosurfaces between the GLN92 amide group of the side chain, N-H of the imidazole ring of HIS64, the hydroxyl group of the THR199 and the OAQ, OAA oxygen atoms, and the H-N^−^ of the sulfonamide group of TPM, respectively. In fact, the bluer the isosurface, the more attractive the interaction—here, these isosurfaces are indicators of the presence of the two N-H…O and one H-O…H-N^−^ hydrogen bonds. It can be seen that these interactions were preserved throughout the course of QM/MM MD, but they were smaller in magnitude (in Figure 6b,c, the isosurfaces corresponding to them are also present, but are greener). Besides, for every snapshot of the QM/MM MD, there was the isosurface between the THR199 backbone N-H and the OAS oxygen of TPM sulfonamide group as well as between the C-H of the backbone or HC-H of the side chain of LEU198 and the OAS oxygen of TPM.

The Quantum Theory of Atoms in Molecules (QTAIM) analysis was performed to quantitatively describe the non-covalent interactions present in the binding pocket of CA I. In this regard, only the properties of the Bond Critical Points (BCPs) corresponding to the closed-shell interactions between the TPM and the binding site of CA I were analyzed and gathered in the decreasing order (with respect to their ED at the BCP) in Table 1. We only extensively examined six the strongest interactions present in each model. Some of the obtained results were not included, due to the chemical nature of the pairs of donor and acceptor atoms and their unclear status in terms of interaction with TPM (whether they are attractive or repulsive). In a snapshot corresponding to 5 ps of MD, it can be seen that THR199 is the amino acid that contributes most to ligand stabilization in the binding pocket through H-O…H-N^−^ and N-H…O=S interactions (OAS oxygen), whose bond energies were estimated to be approximately 8.30 and 2.50 kcal/mol. Besides, GLN92, HIS64, LEU198, and HIS200 significantly contribute to the ligand mode of binding at that instant of time. The estimated bond energies, electronic densities, energy densities, and Laplacian values at the BCP indicate that all of the interactions at a given snapshot can be considered as weak hydrogen bonds—considering the hydrogen acceptors and donors, the presence of the non-conventional C-H…O=S HB between LEU198 and TPM should be noted. In the case of the second snapshot (at 10 ps), it can be seen that the most pronounced interaction between the TPM and the binding site of CA I can be ascribed to HIS64, for which the N-H of the imidazole ring interacts with the OAA oxygen of Topiramate. The second largest bond energy is assigned to H-O…H-N^−^, where the hydroxyl group comes from the THR199 side chain and the H-N^−^ is a part of the sulfonamide group of TPM. Again, HIS64, THR199, GLN92, and LEU198 are the main contributors to the stabilization of the ligand in the binding pocket. As it was the case for the first snapshot, the bond energies of the corresponding non-covalent interactions are rather small and they adopt values ranging from approximately 1.50 to 6.00 kcal/mol. For the third snapshot (at 15 ps) THR199, due to the presence of N-H…O=S (OAS oxygen) and H-O…H-N^−^ secondary bonds, is the amino acid with the most profound impact on the binding mode of the TPM molecule. Furthermore, on the contrary to the snapshots taken at 5 ps and 10 ps, LEU198 interacts with TPM via its CH_2_ group. In conclusion, it is noteworthy that both the second and the third snapshot contain the same amino acids, which were found to provide the greatest stability. The only difference between the first and the next two snapshots is the presence of N-H…O=S interaction with HIS200 residue and the absence of the C=O…H-C contact with GLN92 in the first one. Besides, an inspection of the values presented in Table 1 allows one to tell that physical quantities corresponding to the studied interactions fluctuate during the course of the molecular dynamics, still the underlying interactions reoccur in every examined atom configuration.

The NBO framework was applied to obtain a complementary and more chemically intuitive picture of the studied interactions. The use of NBO analysis allows one to obtain the localized orbitals and to quantitatively investigate the charge transfer from the lone pair orbitals of the donor to the antibonding ($\sigma^{*}$) orbital (estimated at the second-order perturbation theory in the NBO basis) of the acceptor as well as the steric repulsion associated with the interaction and the sum of the attractive and repulsive components to the energy, E_NBO_ [37,38]. The above-mentioned physical quantities associated with the donor–acceptor interactions are gathered in Table 2.

**Table 1:** QTAIM-derived properties at BCPs of six chosen non-covalent interactions taken from snapshots at approximately 5, 10, and 15 ps of the large model in the course of the QM/MM MD. E1 are bond energies based on Espinosa model [39]. Units of gathered quantities are as follows: electron density, $\rho_{BCP}$, is provided in $e\cdot a_{0}^{-3}$ atomic units and the Laplacian of electron density, $\nabla^{2}\rho_{BCP}$, is in $e\cdot a_{0}^{-5}$ units. V_BCP_ stands for BCP potential energy density and H_BCP_ denotes the energy density at the BCP.

Snapshot [ps]	System	BCP	ρ _BCP_	V_BCP_	H_BCP_	$\nabla^{2}$ ρ _BCP_	E1
5	THR199	H-O…H-N^−^	0.0334	−0.0264	0.0027	0.1270	−8.2895
	GLN92	HN-H…O	0.0322	−0.0239	0.0021	0.1128	−7.5059
	HIS64	N-H…O	0.0225	−0.0173	0.0004	0.0722	−5.4147
	THR199	N-H…O=S	0.0104	−0.0079	0.0005	0.0359	−2.4824
	LEU198	C-H…O=S	0.0104	−0.0077	0.0007	0.0360	−2.4119
	HIS200	N-H…O=S	0.0094	−0.0067	0.0002	0.0280	−2.0951
10	HIS64	N-H…O	0.0236	−0.0186	0.0005	0.0782	−5.8295
	THR199	H-O…H-N^−^	0.0234	−0.0172	0.0009	0.0765	−5.4111
	GLN92	HN-H…O	0.0184	−0.0137	0.0002	0.0558	−4.2838
	THR199	N-H…O=S	0.0145	−0.0106	−0.0001	0.0418	−3.3396
	LEU198	C-H…O=S	0.0119	−0.0092	0.0007	0.0424	−2.8745
	GLN92	C=O…H-C	0.0072	−0.0050	0.0012	0.0297	−1.5717
15	THR199	N-H…O=S	0.0225	−0.0157	0.0010	0.0709	−4.9365
	THR199	H-O…H-N^−^	0.0196	−0.0143	0.0004	0.0604	−4.4977
	HIS64	N-H…O	0.0141	−0.0103	0.0001	0.0418	−3.2406
	GLN92	HN-H…O	0.0120	−0.0086	0.0001	0.0350	−2.7019
	GLN92	C=O…H-C	0.0101	−0.0070	0.0001	0.0291	−2.2096
	LEU198	HC-H…O=S	0.0086	−0.0064	0.0010	0.0335	−2.0209

Let us start the discussion with the attractive components to the energy. In the case of the snapshot taken at 5 ps of MD, it can be seen that the largest LP2(O) values are observed for the HN-H…O (GLN92) and H-O…H-N^−^ (THR199)—the magnitudes of the interaction energy associated with that transfer at the second-order perturbational theory are equal to 14.05 and 12.78 kcal/mol, respectively. For both of the above-mentioned interactions, the electron transfer from the LP1(O) to the $\sigma^{*}$ orbital of the acceptor was of the smaller magnitude. Another two interactions of the non-negligible stabilizing effect are ascribed to the presence of HIS64 and HIS200, where the LP1(LP2)(O) $\to \sigma^{*}$ (X—H) energies were equal to 7.15 (1.04) and 1.95 (2.24) kcal/mol, respectively.

The most pronounced non-covalent interactions detected for the snapshot taken at 10 ps were H-O…H-N^−^, N-H…O, HN-H…O, and N-H…O=S that stems, respectively, from the interaction of TPM with THR199, HIS64, GLN92, and THR199. For the above-mentioned interactions, the charge transfer associated E_NBO_ from LP1 (LP2) was equal to 3.41 (6.99) kcal/mol for H-O…H-N^−^, 6.32 (1.39) kcal/mol for N-H…O, 4.64 (2.10) kcal/mol for HN-H…O, and 2.88 (3.81) kcal/mol for N-H…O=S. It can be seen that the THR199-involving interactions were significantly weakened and strengthened, respectively, when compared to the E_NBO_ values seen for the first snapshot. Also noteworthy is the weakening of the HN-H…O interaction (GLN92) and the preservation of the attractive component to the energy of the N-H…O interaction (HIS64).

It can be seen for each studied model that the E_NBO_ energies associated with the interactions between TPM and the HIS64, THR199, and GLN92 (HN-H…O) amino acids are the most pronounced ones. The charge transfer energies related to the weak hydrogen bonds (C=O…H-C, C-H…O=S, and HC-H…O=S) were smaller in magnitude, which was due to the lower electronegativity of the carbon atom and, thus, the lowered acidity of the corresponding proton.

Thus, in the light of the results obtained here, the C=O…H-C (GLN92), HC-H…O=S (LEU198), and C-H…O=S (LEU198) interactions cannot be regarded as attractive in nature, because they are repulsive.

To conclude, it should be noted that the NBO allowed us to quantify the stabilization corresponding to the particular interactions under study; moreover, it enabled us to discern between attractive and repulsive interactions.

**Table 2:** Natural Bond Orbitals (NBO) interaction and steric repulsion energies estimation between the lone pairs of the oxygen acceptor atom and the antibonding $\sigma^{*}$ (X–H) or bonding σ (X–H) orbital of the amino acids of the CA I binding site (large model) and the Topiramate ligand. **E_NBO_** is defined as a sum of attractive and repulsive components to the energy. All energies are provided in kcal/mol.

Snapshot [ps]	System	Interacting Pair	LP1(O) $\to \sigma^{*}$ (X–H)	LP2(O) $\to \sigma^{*}$ (X–H)	σ (X–H) → LP1(O)	σ (X–H) → LP2(O)	E_NBO_
5	THR199	H-O…H-N^−^	−3.95	−12.78	2.69	9.81	−4.23
	GLN92	HN-H…O	−5.51	−14.05	2.45	9.51	−7.60
	HIS64	N-H…O	−7.15	−1.04	5.24	1.56	−1.39
	THR199	N-H…O=S	−0.99	−0.20	0.86	0.22	−0.11
	LEU198	C-H…O=S	−0.63	−0.39	2.11	0.18	1.27
	HIS200	N-H…O=S	−1.95	−2.24	0.73	0.65	−2.81
10	HIS64	N-H…O	−6.32	−1.39	4.51	1.75	−1.45
	THR199	H-O…H-N^−^	−3.41	−6.99	2.00	4.61	−3.79
	GLN92	HN-H…O	−4.64	−2.10	2.88	1.90	−1.96
	THR199	N-H…O=S	−2.88	−3.81	1.63	1.40	−3.66
	LEU198	C-H…O=S	−0.93	−0.15	2.95	—	1.87
	GLN92	C=O…H-C	−0.35	−0.14	0.91	0.50	0.92
15	THR199	N-H…O=S	−6.68	−4.49	3.41	1.89	−5.87
	THR199	H-O…H-N^−^	−3.94	−4.52	2.24	3.06	−3.16
	HIS64	N-H…O	−3.36	−1.22	2.27	0.64	−1.67
	GLN92	HN-H…O	−3.74	−0.40	1.82	0.27	−2.05
	GLN92	C=O…H-C	−0.41	−0.93	0.69	1.09	0.44
	LEU198	HC-H…O=S	−0.75	—	1.86	—	1.11

### 2.2. Analysis of Non-Covalent Interactions and Dative Covalent Bonds of a Small Model

**Figure 7:**
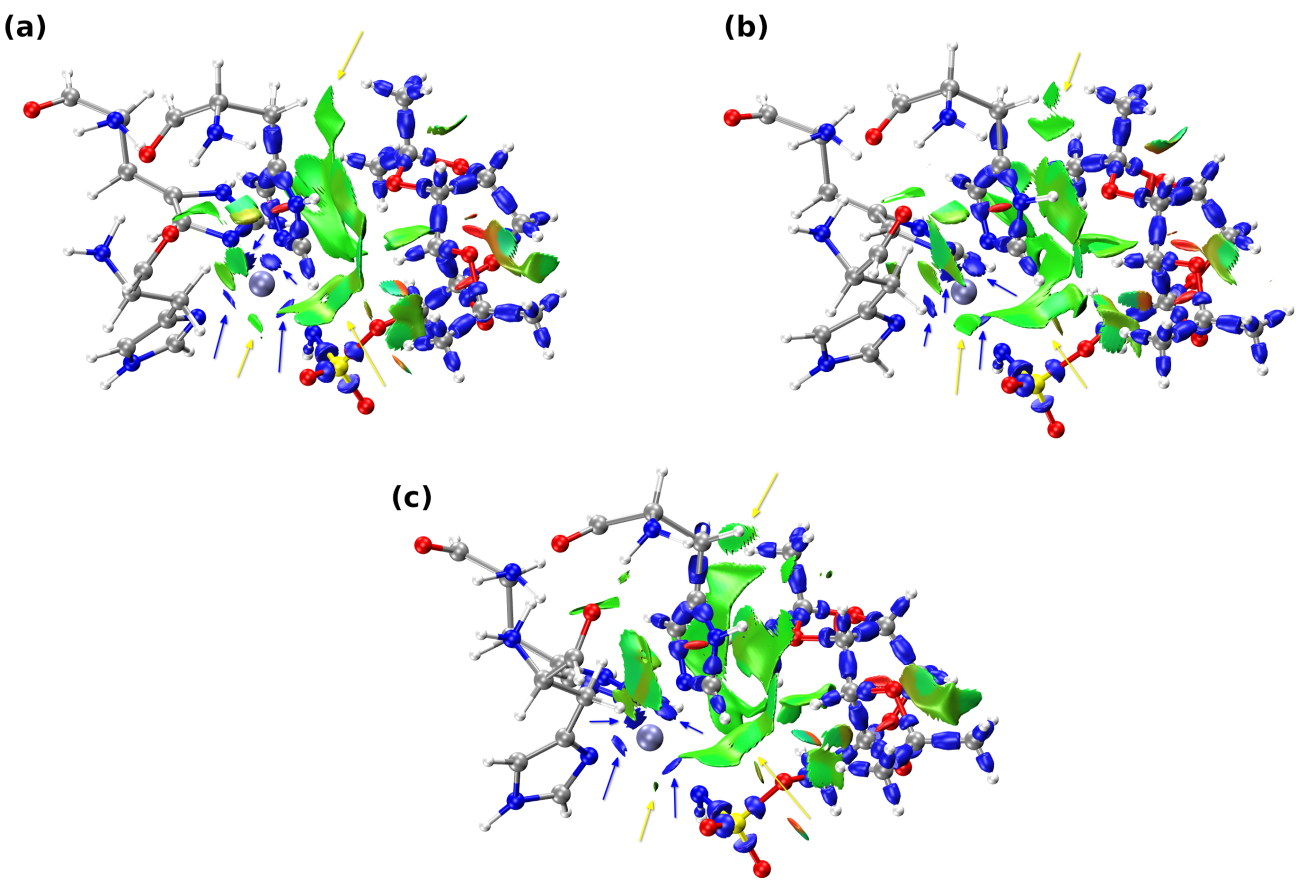
Visualization of the non-covalent interactions, covalent and dative covalent bonds of a small model using the IRI method. Structures were taken from the course of QM/MM Molecular Dynamics. (**a**) Snapshot at 5 ps, (**b**) snapshot at 10 ps, and (**c**) snapshot at 15 ps. All atoms are depicted using the CPK representation. Color coding: grey—carbon, white—hydrogen, red—oxygen, blue—nitrogen, yellow—sulfur, and zinc—iceblue. Selected isosurfaces color coding in the ED range: −0.035 < sign(λ_2_)ρ < 0.02 a.u.

Zinc coordination and the neighboring non-covalent interactions between the TPM ligand and the three histidine molecules (HIS94, HIS96, and HIS119) were investigated using Interaction Region Indicator (IRI) [35] (via analyzing selected isosurfaces), which can be considered as a redefined Reduced Density Gradient (RDG) [40] method, to simultaneously show the covalent and non-covalent interactions. In fact, IRI differs from the RDG only by the constant prefactor and the parameter (a = 1.1 for IRI and a = 4/3 for RDG) present in the denominator, which scales the electronic density. Analogous to the RDG method, one may discern the nature of the detected interactions using the *sign*(λ_2_)ρ. On the basis of this quantity, the isosurfaces are colored differently—the more attractive the interaction, the more blueish is its isosurface, whereas, when the interaction is repulsive, the isosurface assumes brown to red color. The visualization of the obtained results using the IRI method is presented in Figure 7. The yellow arrows present in Figure 7 indicate the presence of the van der Waals based non-covalent interactions, whereas blue arrows point to the isosurfaces corresponding to the dative covalent bonds. Due to the proximity of the TPM ligand to HIS94, most of the detected non-covalent interactions derive from contacts between them—of particular importance, here, are the interactions between the OAF and OAN oxygens from the TPM and imidazole as well as acidic hydrogen (C-H) of the imidazole ring, respectively. Besides, there are a plethora of C-H…H-C and C-H…imidazole contacts, that may have a non-negligible impact on the ligand position in the binding site. Furthermore, the IRI analysis detected interactions of van der Waals type between TPM and imidazole and CH_2_ of HIS119 (these isosurfaces were shrinked for clarity - they were obscuring the isosurfaces related to coordination bonds) as well as four dative covalent bonds (blue isosurfaces) in each studied model—they were preserved in each examined snapshot.

In general, it can be seen that the IRI analysis applied to three arrangements of atoms (Figure 7) has provided us with evidence that the ligand position in the CA I binding pocket is maintained during the MD simulations. It is evident that, besides strong, dative covalent bonds, the non-covalent interactions (mainly between HIS94 and TPM) are changing marginally.

The QTAIM method was used to quantitatively examine the dative covalent bonds present in the small model (data corresponding to the QTAIM analysis of the small model is presented in Table 3). It can be seen that the values of the electron density at detected BCPs change from snapshot to snapshot—for example: Zn-N (HIS94) and Zn-N^−^ are characterized by the largest values of the ρ at BCPs in the first snapshot, whereas the most pronounced values of ED at BCPs of second and third snapshots can be ascribed to the presence of TPM, HIS94 and HIS119, TPM molecules, respectively. Further inspection of the data gathered in Table 3 shows the partially covalent nature of the examined dative covalent bonds, which is due to the negative values of H_BCP_ and positive ones of $\nabla^{2}\rho_{BCP}$. As it was the case in the IRI analysis, the QTAIM results supplement us with the evidence that the detected coordinate bonds were stable throughout the course of the molecular dynamics simulations.

In summary, the detachment of the H atom from the sulfonamide group allows for TPM to bind to the zinc cation via the dative-covalent bond, which acts like an anchor for the inhibitor. Moreover, one can easily observe that the position of the ligand in the binding pocket of CA I changes accordingly with the fluctuating shape of the binding cavity. Attention should be paid to the neighboring amino-acids that provide significant stabilization and hold the ligand firmly in the CA I active site—it was indicated that these non-covalent interactions are of a weak kind and their strength changes in a function of time, nevertheless, in general, remaining attractive in nature for every examined snapshot.

### 2.3. Topiramate-CA I Binding Pocket Interaction Energy Estimation and Its Decomposition

The interaction energy decomposition allowed for revealing the nature of interactions between the TPM ligand and the amino acids in the most pronounced way involved in its binding, namely: HIS94, THR199, GLN92, LEU198, HIS200, SER65, and HIS64. In Table 4, the energy components to the E_int_ as well as share of each component with respect to the total attractive interaction energy were gathered.

It can be seen that the shares of each component to the total attractive E_int_ does not significantly change from one snapshot to the other (they diverge from each other within 5 percentage points). In the case of the first snapshot (5 ps), the interaction energy depends, to the largest extent, on the E_disp_ and E_elst_ that constitute up to approximately 42% and 37% of the total attractive energy, respectively. Furthermore, it can be seen that the magnitude of E_elst_ assumes an exceptionally large value when compared to the second and third snapshot. This observation can be supported by the results of the QTAIM and NBO analyses of the large model (see previous subsection), where two weak HBs between TPM and THR199 as well as GLN92 amino acids provide substantially larger stabilization than their counterparts at 10 and 15 ps—the weak hydrogen bonds are characterized by large electrostatic components to the interaction energy [11,41]. The comparison of the dispersion energy contributions between the snapshots causes it to be possible to state that its share to the total attractive interaction energy rises for the second and third snapshot; however, its magnitude decrease steadily from first to third. The first thing is obviously related to the decreased magnitude of E_elst_ components for 10 and 15 ps, whereas the second can be explained with reference to the relative position of the binding site amino acids to the ligand molecule (in general, the distance between the ligand and amino acids is larger)—it is observed (especially for the third snapshot) that the E_exch_, E_ind_, and E_disp_ significantly decreased in magnitude. Under these circumstances, one should have in mind their dependence on the interatomic separation (R). It is known that the exchange energy decreases exponentially with the **R** increase, whereas induction as well as dispersion decrease as **R**^−n^ (where n is usually equal to 3 and 6, respectively).

To conclude, dispersion and electrostatics energy components are the main contributions to the interaction energy of the examined systems and their total contribution to the attractive E_int_ reaches approximately 80%.

## 3. Materials and Methods

### 3.1. Molecular Dynamics in a Hybrid QM/MM Scheme

The initial structure of the complex of Carbonic anhydrase I—Topiramate (TPM) was taken from the Protein Data Bank (PDB) database (PDB entry: 3LXE) [14]. Initially, the model of the protein (single polypeptide chain) was neutralized, supplemented with missing hydrogen atoms, and embedded into an 67.103 × 65.534 × 72.989 orthorhombic box of water molecules using AmberTools21 [42,43]. The Amber ff14SB [44] and GAFF [45] force fields for protein and non-protein residues were employed, respectively, whereas TIP3P was used as a water model [46]. The energy minimization of the initial structure was carried out using the steepest descent algorithm (10,000 steps). The equilibration of the protein model consisted of NVT (Langevin thermostat) and NPT (Langevin thermostat, Berendsen barostat, p = 1 atm) phases, which lasted for 0.2 and 0.8 ns, respectively, with the temperature increased in a gradual manner, from 0 to 298.15 K over 140 ps (after that, the temperature was kept equal to 298.15 K). The aforementioned force fields and water model were used throughout the course of the QM/MM Molecular Dynamics as well. The non-bonded interactions were cut off at 10 Å. The parametrization of the binding metal site (three histidines and ligand) of the CA I was performed with usage of the MCPB.py [47] software incorporated in AmberTools21. The calculations of the QM part were performed using the Gaussian plane-wave (GPW) method [48,49] and the Density Functional Theory (DFT) [50,51], which are implemented in the CP2K code [52,53]. The core electrons were represented using Goedecker–Teter–Hutter (GTH) pseudopotentials [54,55] and the molecular orbitals were expanded using molecularly optimized DZVP-MOLOPT-GTH basis sets [56]. The plane-wave cutoff was set to 500 Ry. The gradient-corrected exchange–correlation functional of Perdew, Burke, and Ernzerhof and Grimme dispersion corrections with a Becke–Johnson damping function, denoted as D3(BJ), were used [57,58,59]. The effects of the periodic boundary conditions in the QM/MM scheme were included using the multigrid approach with the Gaussian expansion of electrostatic potential (GEEP). The polarization of the QM region due to the presence of the classical charges of the rest of the protein and the water environment was included via the so-called electrostatic embedding. The Nośe–Hoover chain thermostat [60,61] was employed in order to maintain the ergodicity of the dynamics and provide the canonical distribution. The system was decomposed into two regions (QM and MM) and the temperature in both was set to 298.15 K. The simulation was carried out in the cubic box with a = 23 Å. The trajectory was collected for approximately 17 ps and the initial 2 ps of the trajectory was taken as the equilibration phase, whereas the time step was set to 0.5 fs. The simulation facilitated a comprehensive description of the molecular features in the binding pocket. The snapshots from the trajectory were taken at ca. 5 ps, 10 ps, and 15 ps of the production run. The QM and MM boundary of the protein was drawn along the C(alpha)-C(beta) (Integrated Molecular Orbital Molecular Mechanics (IMOMM) [62] link type was used) bonds of the following amino acids: His119, Thr199, His200, His96, His64, Ser65, His94, Gln92, and His67. Moreover, two water molecules (in close proximity to the ligand molecule throughout the course of all MD), as well as Topiramate and the zinc cation were treated as the QM region. The above-mentioned simulations were performed using the AMBER 2020 and CP2K version 8.2 set of codes [52,53,63].

### 3.2. Density Functional Theory (DFT) and Wavefunction Analysis

Density Functional Theory (DFT) [50,51] was applied to systems extracted from the QM/MM MD. We split the binding site into two different systems:

(i) Larger one, which included the ligand molecule as well as the nearest amino acids (within 3.5 Å) with two water molecules;
(ii) Smaller one, which included ligand molecule as well as zinc atoms and the amino acids involved in its coordination.

The combination of the ωB97X exchange–correlation functional [64] with Grimme D3 dispersion corrections as well as a Becke–Johnson damping function (D3-BJ) [58,59,65] and def2-SVP basis set [66] was employed to obtain the wavefunction of the larger systems. The smaller systems’ wavefunctions were obtained using the same exchange–correlation functional and dispersion corrections, but with a larger basis set: def2-TZVPD [66,67]. This part of the calculations was performed with the usage of ORCA 5.0.3 software [68]. Then, the Quantum Theory of Atoms in Molecules (QTAIM) [33] formalism was used to analyze the electron density and its Laplacian at bond critical points (BCPs). The obtained information provided us with evidence of the kind of interactions present in the studied systems, e.g., the presence of BCP enabled us to discuss the covalent and non-covalent interactions [69]. We can discern between these two kinds of interactions on the basis of the values of the ED and its Laplacian at the BCP; furthermore, the values of the V_BCP_ (potential energy density) allowed for an estimation of the interaction energies of particular contacts. The QTAIM analysis was performed in the non-equilibrium structures. However, the usual nomenclature, associated with standard equilibrium–structure QTAIM calculations, was applied for convenience, following the practice found in the literature [70,71]. In order to obtain further insight into the electronic structure, the Interaction Region Indicator (IRI) and Independent Gradient Model based on Hirshfeld partitioning (IGMH) analyses were performed. In the case of both analyses, the Multiwfn 3.8 [72] served as the software of choice. Furthermore, the NBO 6.0 program (coupled with ORCA 5.0.3) [68] was used to analyze the network of interactions from the Natural Bond Orbital (NBO) theory [34,73] viewpoint. It is important to underline the justification for using a smaller basis set for a larger system—our version of NBO 6.0 [74] prevented us from performing calculations at the more robust level of theory due to the basis set and memory limits imposed by the developers on the binary distribution.

The visualization of the obtained results was prepared with the assistance of the VMD 1.9.3 [75] and SAMSON [76] suite of programs.

### 3.3. Symmetry-Adapted Perturbation Theory (SAPT) Decomposition of the Interaction Energy

Symmetry-Adapted Perturbation Theory (SAPT) [36,77] was employed to decompose the ligand–protein interaction energy into physically meaningful contributions. The SAPT scheme was applied at the sSAPT0/jun-cc-pVDZ [78] (scaled SAPT0) level of the theory and the density fitting with the auxiliary basis sets of jun-cc-pVDZ-RI as well as jun-cc-pVDZ-JKI was used to accelerate the calculations and approximate the two-center integrals. The ligand and the neighbouring amino acids were treated as two separate monomers to fulfill the requirements of the counterpoise correction method of Boys and Bernardi [79]. This part of the study was performed using Psi4 1.3.2 [80] software.

## 4. Conclusions

Molecular dynamics in the hybrid scheme (QM/MM) was employed to investigate the role and impact of non-covalent interactions on the binding mode of Topiramate (TPM). In order to prepare a comprehensive description of the binding site reorganization upon the ligand presence and dynamical nature of the complex, two sets of models were extracted from the protein–ligand complex and they were denoted as large and small models in the study. For each of the models, three snapshots from the QM/MM MD production run were extracted and the network of the non-covalent interactions was analyzed using IGMH, IRI, QTAIM, and NBO electron-density-based methods. Our investigations have shown the following:

(i) The physical quantities at BCPs and acceptor–donor properties of the studied interactions (as well as the donors of interactions) were reorganizing throughout the course of the MD; nonetheless, the ligand was firmly kept in the binding site for all the simulation time;
(ii) The application of IGMH, IRI, QTAIM, and NBO methods allowed for obtaining a complementary and detailed picture of the particular secondary bonds of interest—notably, the NBO analysis enabled us to reveal the nature (attractive or repulsive) of the examined non-covalent interactions;
(iii) The energy decomposition supported the observations about the binding site reorganization from other analyses and it showed that the most pronounced components to the interaction energy of the TPM-CA I binding site are electrostatics and dispersion.

Therefore, the most significant non-covalent interactions in the system studied were found: THR199 (H-O…H-N${}^{-}$ as well as N-H…O=S), GLN92 (HN-H…O), and HIS64 (N-H…O).

The applied electronic-structure based methods, could be very useful in rational drug design. They enable us to provide a detailed picture of the non-covalent interactions responsible for the ligand conformation as well as its stabilization in the binding pocket. Therefore, we can control and change the strength of interactions. In practice, we could modulate the physico-chemical properties responsible for the biological effect. Furthermore, QM-based methods, which can take into account the presence of σ-hole and π-hole kind of interactions could provide the accurate description necessary in successful drug design.

## Figures and Tables

**Table 3:** QTAIM-derived properties at BCPs of Zn-N interactions (dative covalent bonds) taken from snapshots at approximately 5, 10, and 15 ps of the small model in the course of the QM/MM MD. Units of gathered quantities are as follows: electron density, $\rho_{BCP}$, is provided in $e\cdot a_{0}^{-3}$ atomic units and the Laplacian of the electron density, $\nabla^{2}\rho_{BCP}$, is in $e\cdot a_{0}^{-5}$ units. V_BCP_ stands for BCP potential energy density and H_BCP_ denotes the energy density at the BCP.

Snapshot [ps]	Acceptor	BCP	ρ _BCP_	V_BCP_	H_BCP_	$\nabla^{2}$ ρ _BCP_
5	TPM	Zn-N^−^	0.0987	−0.1583	−0.0271	0.4166
	HIS96	Zn-N	0.0787	−0.1186	−0.0152	0.3529
	HIS119	Zn-N	0.0867	−0.1364	−0.0197	0.3875
	HIS94	Zn-N	0.1024	−0.1769	−0.0292	0.4738
10	TPM	Zn-N^−^	0.0979	−0.1584	−0.0261	0.4251
	HIS96	Zn-N	0.0794	−0.1190	−0.0157	0.3504
	HIS119	Zn-N	0.0757	−0.1092	−0.0143	0.3223
	HIS94	Zn-N	0.0807	−0.1206	−0.0168	0.3482
15	TPM	Zn-N^−^	0.0919	−0.1449	−0.0222	0.4017
	HIS96	Zn-N	0.0851	−0.1337	−0.0183	0.3880
	HIS119	Zn-N	0.0938	−0.1538	−0.0238	0.4250
	HIS94	Zn-N	0.0749	−0.1083	−0.0139	0.3221

**Table 4:** Interaction energies between TPM ligand and the amino acids in the most pronounced way involved in its binding. The calculations were performed at the sSAPT0/jun-cc-pVDZ (**Total**^a^) level of theory; energies are provided in kcal/mol. The percentages provided in the Table correspond to share of each particular component in the total attractive interaction energy.

Snapshot	E_elst_	%	E_exch_	E_ind_	%	E_disp_	%	Total^a^
5 ps	−41.710	37.00	77.113	−24.036	21.30	−47.009	41.70	−35.642
10 ps	−33.281	34.10	65.338	−20.362	20.90	−43.874	45.00	−32.179
15 ps	−27.195	32.80	46.730	−18.667	22.50	−37.056	44.70	−36.188

## Data Availability

Data is contained within the article.

## Abbreviations

The following abbreviations are used in this manuscript:

BCP	Bond Critical Point
BOMD	Born–Oppenheimer Molecular Dynamics
BSSE	Basis Set Superposition Error
CPMD	Car–Parrinello Molecular Dynamics
DFT	Density Functional Theory
ED	Electron density
EDA	Energy Decomposition Analysis
HB	Hydrogen Bond
IGM	Independent Gradient Model
IGMH	Independent Gradient Model based on Hirshfeld partitioning
IMOMM	Integrated Molecular Orbital Molecular Mechanics
IRI	Interaction Region Indicator
MD	Molecular Dynamics
MEP	Molecular Electrostatic Potential
NBO	Natural Bond Orbitals
NCI	Non-Covalent Interaction
NMR	Nuclear Magnetic Resonance
RDG	Reduced Density Gradient
QM/MM	Quantum Mechanics/Molecular Mechanics
QTAIM	Quantum Theory of Atoms in Molecules
SAPT	Symmetry-Adapted Perturbation Theory
WBI	Wiberg Bond Index
